# Effect of the Age-Adjusted Charlson Comorbidity Index on the Survival of Esophageal Squamous Cell Carcinoma Patients after Radical Esophagectomy

**DOI:** 10.3390/jcm11226737

**Published:** 2022-11-14

**Authors:** Jianyu Song, Yulan Lin, Juwei Zhang, Shuang Liu, Jinsong Zhou, Qiaoyan Zeng, Zheng Lin, Rong Fu, Minglian Qiu, Zhijian Hu

**Affiliations:** 1 Department of Epidemiology and Health Statistics, School of Public Health, Fujian Medical University, Fuzhou 350122, China; 2 Department of Thoracic Surgery, The First Affiliated Hospital of Fujian Medical University, Fuzhou 350005, China; 3 Key Laboratory of Ministry of Education for Gastrointestinal Cancer, Fujian Medical University, Fuzhou 350122, China

**Keywords:** age-adjusted Charlson comorbidity index, esophageal squamous cell carcinoma, esophagectomy, postoperative, prognosis

## Abstract

We aimed to investigate whether the age-adjusted Charlson comorbidity index (ACCI) can predict the postoperative overall survival (OS) and cancer-specific survival (CSS) of esophageal squamous cell carcinoma (ESCC) patients. Between 1 July 2015 and 31 July 2021, a retrospective cohort study was conducted among patients with primary ESCC who underwent radical esophagectomy. A total of 352 patients were included, with median age of 63.00 (IQR (interquartile range) 56.00–68.00). The patients were divided into low (n = 300) and high (n = 52) ACCI groups based on the optimal cut-off value of 5 points. Chronic pulmonary disease (38.4%) was the most common comorbidity. The results of the multivariate Cox regression showed that the ACCI (HR = 1.63, 95%CI: 1.04–2.56), tumor size (HR = 1.67, 95%CI: 1.05–2.66), pTNM (II vs. I, HR = 4.74, 95%CI: 1.82–12.32; III vs. I, HR = 6.08, 95%CI: 2.37–15.60), and postoperative chemotherapy (HR = 0.60, 95%CI: 0.40–0.91) were significantly associated with the OS. Furthermore, the ACCI, tumor size, pTNM, and postoperative chemotherapy were also significantly associated with the CSS. Interactions were identified between the ACCI and postoperative chemotherapy, pTNM stage, and tumor size in relation to the OS and CSS. In conclusion, the ACCI may be an independent prognostic factor affecting the long-term prognosis of patients after radical esophagectomy.

## 1. Introduction

Esophageal cancer, ranking with the sixth-highest mortality rate of all cancers, caused 544,000 deaths in 2020 [1]. In particular, in regions with a high incidence of esophageal cancer, including Eastern Asia, Southern Africa, Eastern Africa, and Northern Europe, esophageal cancer is a severe burden on public health [2,3,4]. Esophageal squamous cell carcinoma (ESCC) and esophageal adenocarcinoma (ECA) are the two main types of esophageal cancer worldwide. In China, ESCC is the most common type of esophageal cancer, accounting for more than 90% of the total cases, with more than 200,000 new cases occuring every year [5,6]. Surgery and chemotherapy are the two main treatments for patients with local and advanced esophageal cancer. In recent decades, the survival rate has been improved due to improvements in the management and treatment of esophageal cancer patients. However, the general outcome remains very poor with respect to the overall 5-year survival rates (~10%) and 5-year post-esophagectomy survival rates (~15–40%) [7]. More importantly, given the increase in life expectancy in China, the incidence of esophageal cancer among elderly patients with comorbidities [8], who are more likely to have postoperative complications, including death, has continued to increase.

In 1987, Charlson developed the Charlson comorbidity index (CCI) to evaluate preoperative comorbidities in order to predict mortality [9]. The index is a weighted measure that incorporates 19 different medical categories, with each category being weighted according to its impact on mortality. In 1994, Charlson et al. established a new scoring system, the age-adjusted CCI (ACCI), to predict operative mortality more accurately [10]. As a widely used score for quantifying age and a variety of comorbidities, the ACCI is regarded as a useful predictor of mortality in the context of several cancers, including ovarian cancer, prostate cancer, pancreatic cancer, and colorectal cancer [11,12,13,14,15].

As far as we know, only one recent Japanese study reported that the ACCI may be considered as a significant predictor of both the overall survival (OS) and recurrence-free survival in patients with esophageal cancer after radical esophagectomy [16]. However, this study had a small sample size (n = 122), and the results have not been confirmed in other study populations. Hence, the conclusion remains controversial. Therefore, the current study mainly aimed to investigate the associations between the ACCI and long-term prognosis of patients with esophageal cancer who underwent radical esophagectomy.

## 2. Materials and Methods

### 2.1. Study Design and Participants

A retrospective cohort study was conducted between 1 July 2015 and 31 July 2021 in the First Affiliated Hospital of Fujian Medical University, Fuzhou City, China. The inclusion criteria were as follows: (A) patients newly diagnosed with primary ESCC, who (B) had lived in Fujian Province for the past 10 years. The exclusion criteria were as follows: (A) patients with second primary cancer; (B) recurrent or metastasized cancer cases; (C) and patients who underwent preoperative radiotherapy or chemotherapy. More details about the study design can be found in our previous publication [17].

### 2.2. Data Collection

Upon hospital admission, the eligible patients participated in face-to-face interviews conducted by trained interviewers using a self-developed questionnaire. Demographic characteristics, such as age and gender, were collected through the questionnaire. Clinical data, including the tumor size, pathology tumor node metastasis (pTNM) stage, degree of differentiation, postoperative chemotherapy, postoperative complications, and comorbidity diseases were extracted from electronic medical records. The tumor staging was evaluated by a pathologist according to the 8th edition of the American Joint Committee on Cancer (AJCC) TNM staging manual [18]. After discharge from the hospital, the patients were followed up by phone call at a frequency of once every three months in the first year and then once every six months in the second and subsequent years. The end of the follow-up was set as 31 July 2021 or death, whichever occurred first. The survival status and death date were obtained from the death registration records of the local centers for disease control (CDC).

### 2.3. Surgical Procedure and Other Treatments

All the patients received conventional thoracoscopic radical resection of the esophageal cancer, such as two-incision or three-incision intrathoracic esophagectomy and esophagogastrostomy, as well as local lymph node dissection. Postoperative chemotherapy was performed mainly based on the histopathology of the resected esophagus, symptoms, signs, and all the necessary auxiliary examination results. All these treatments were conducted in accordance with the Chinese national Guidelines for the Diagnosis and Treatment of Esophageal Cancer [19].

### 2.4. Definition of the Comorbidities

The pathologies of the Charlson comorbidities were identified through the ICD-10 codes of the discharge diagnoses and long-term disease, specific medical procedures, and the reimbursement of specific medications in the 12 months before the patients’ inclusion [20,21]. The ACCI score was calculated on the basis of the CCI, and the risk increased by one point for every decade over the age of 40 (e.g., 50–59 years old, 1 point; 60–69 years old, 2 points, etc.) [10]. In the current study, esophageal cancer was not regarded as a cancer comorbidity, because the scores associated with other malignant tumors may better predict the CSS in patients who undergo esophagectomy [22].

### 2.5. Survival Data

The follow-up time was calculated from date of surgery until date of death or the end of the follow-up, whichever occurred first. The overall survival (OS) was defined as the time interval from surgery to the date of death. The cancer-specific survival (CSS) was defined as the time interval from surgery to date of death due to ESCC.

### 2.6. Statistical Analysis

Categorical variables were presented as the frequency with the percentage and were compared using a chi-square test. Continuous variables with a non-normal distribution were presented as the median and interquartile range (IQR) and compared using the Mann–Whitney U test. The optimal cut-off values of the ACCI and tumor size were determined through X-Tile (Version 3.6.1, Yale University, New Heaven, CT, USA) [23]. In particular, the optimal cut-off values of the ACCI and tumor size were 5 points and 3.3 cm, respectively. Kaplan–Meier curves were used to exhibit the OS and CSS, while the differences were compared by the log-rank method. Cox proportional hazard models were used to explore the factors related to survival. The variables included in the Cox regression model were the age group (<65, ≥65 years), gender (female, male), ACCI (<5, ≥5), tumor size (<3.30, ≥3.30 cm), pTNM (I, II, III), degree of differentiation (high/medium, poor), postoperative chemotherapy (yes, no), and postoperative complications (yes, no). It is worth mentioning that the age group was not adjusted in the multivariate Cox regression model, because the ACCI already considered age during the index calculation. All the statistical analyses were carried out using the SPSS software (Version 20.0, SPSS Inc., Chicago, IL, USA).

The predictive ability of the ACCI for prognosis were evaluated using the C-index [24]. C = 1 means a perfect prediction accuracy, while C = 0.5 means a random prediction [25]. A previous study demonstrated that a C-index with value of 0.7 indicates a good discrimination ability [26]. In order to further measure the predictive value of the ACCI, we also established a nomogram based on the Cox proportional hazard regression model and calculated the risk scores of the independent prognostic factors (R software, Version 4.0.3, R Project, Vienna, Austria).

## 3. Results

### 3.1. Comorbidity

A total of 352 patients with primary ESCC were included in this study between 1 July 2015 and 31 July 2021. The distributions of the comorbidity diseases are listed in Table 1. Chronic pulmonary disease was the most common comorbidity (n = 135, 38.4%), followed by peripheral vascular disease (n = 100, 28.4%) and mild liver disease (n = 65, 18.5%).

### 3.2. Demographic and Clinical Baseline Characteristics

Table 2 presents the demographic and clinical baseline characteristics of all the patients. More than half of the patients were aged <65 years (63.9%), with a median age of 63.00 years (IQR = 56.00–68.00 years). The majority of the patients were male (74.1%), at the pTNM III stage (42.3%), had a high/medium degree of differentiation (65.1%), and had undergone postoperative chemotherapy (69.0%) and had postoperative complications (59.4%). The patients were divided into two ACCI groups: the ACCI < 5 group (n = 300, 85.2%) and ACCI ≥ 5 group (n = 52, 14.8%). Patients in the high ACCI group were older than those in the low ACCI group (*p* < 0.001). By the end of the follow-up on 31 July 2021, the overall median follow-up time was 23.37 months (IQR = 14.08–33.45).

### 3.3. Survival Analysis

**Figure 1:**
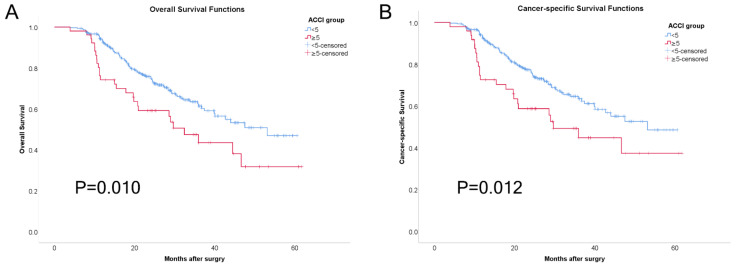
Kaplan–Meier curves for overall survival (**A**) and cancer-specific survival (**B**) for the different ACCI groups.

Figure 1 shows the Kaplan–Meier curves for both the OS and CSS. During the follow-up period between 1 July 2015 and 31 July 2021, the survival rates in the low ACCI group were superior to those of the high ACCI group in terms of both the OS (69.7 vs. 48.1%, *p* = 0.010) and CSS (71.1 vs. 51.0%, *p* = 0.012).

Associations between the potential prognostic factors and OS among patients with ESCC are presented in Table 3. The results of the multivariate Cox regression analysis showed that a higher ACCI (HR = 1.63, 95% CI: 1.04–2.56) and larger tumor size, at ≥3.30 cm (HR = 1.67, 95% CI: 1.05–2.66), were associated with a near two-fold-higher risk of overall mortality. In comparison to pTNM stage I, patients with stage II (HR = 4.74, 95% CI: 1.82–12.32) and stage III (HR = 6.08, 95% CI: 2.37–15.60) had a more than 4–6-times-higher risk of mortality. Meanwhile, postoperative chemotherapy was negatively associated with the risk of overall mortality (HR = 0.60, 95% CI: 0.40–0.91).

Table 4 shows the results of the analysis of the associations between the potential prognostic factors and CSS. Several factors were associated with cancer-specific mortality, including the higher ACCI group (HR = 1.75, 95%CI: 1.08–2.82), a larger tumor size at ≥ 3.30 cm (HR = 1.66, 95%CI: 1.03–2.69), pTNM stage II (HR = 4.70, 95%CI: 1.80–12.23), and pTNM stage III (HR = 5.99, 95%CI: 2.33–15.40). Postoperative chemotherapy was negatively associated with the risk of cancer-specific mortality (HR = 0.57, 95%CI: 0.37–0.87).

**Figure 2:**
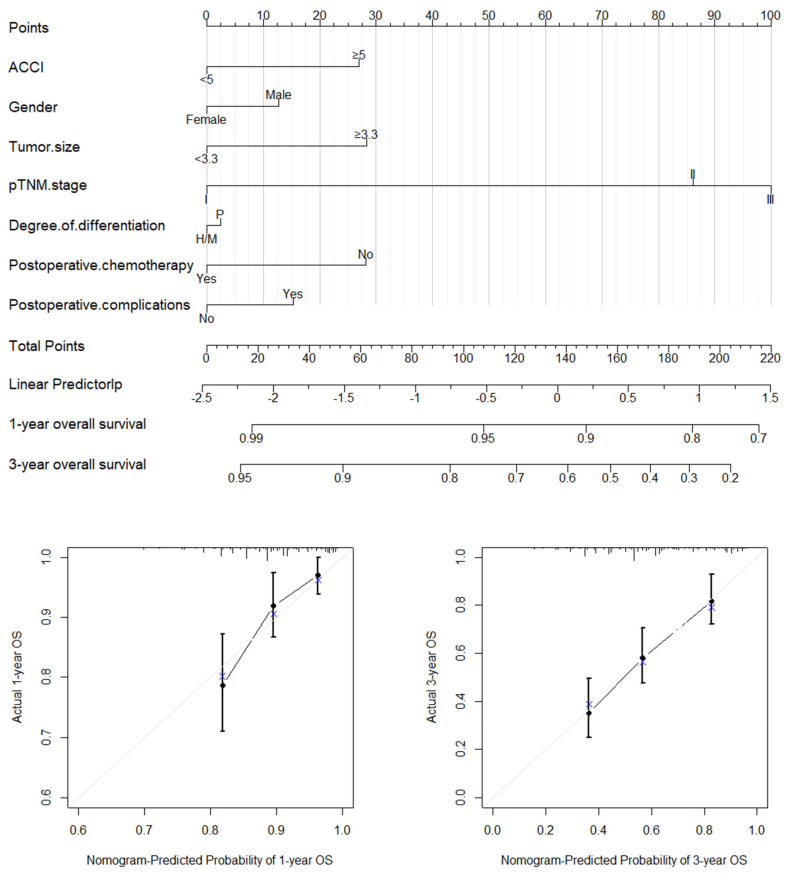
Nomogram plot and calibration curves of the 1−year and 3−year OS.

**Figure 3:**
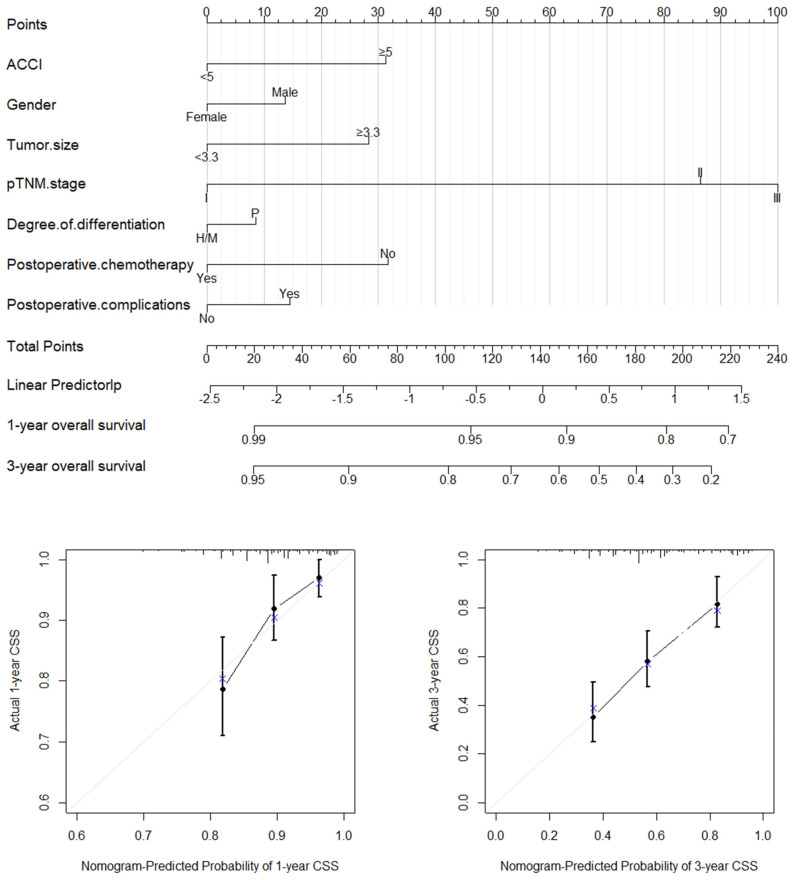
Nomogram plot and calibration curves of the 1−year and 3−year CSS.

Based on the significant prognostic factors identified through multivariate Cox regression, we established nomograms for both the OS and CSS. Figure 2 and Figure 3 present the nomogram plots and calibration curves of the 1-year and 3-year OS and CSS. The C-index was 0.712 (95%CI: 0.664–0.760) for the OS and 0.716 (95%CI: 0.667–0.765) for the CSS, which indicated a good discrimination. We also performed a nomogram validation after 1000 bootstrapping calculations. All the calibration curves of the 1-year and 3-year OS and CSS were close to the diagonal line.

Several interactions were found between the ACCI and other prognostic factors (chemotherapy, pTNM stage, tumor size, all *p* ≤ 0.05; see Supplementary Tables S1 and S2). In order to further explore these interactions, we conducted subgroup analyses. In the subgroup of patients with tumor sizes of < 3.3 cm, a higher ACCI was significantly associated with the OS (HR = 4.40, 95%CI: 1.75–11.08) and CSS (HR = 6.13, 95%CI: 1.93–19.42). No significant results were observed in the subgroup of patients with tumor sizes of ≥ 3.3 cm (Supplementary Figure S1 and Supplementary Figure S2). Additionally, in the pTNM = I group (OS, HR = 9.30, 95%CI: 1.16–74.75; CSS, HR = 9.30, 95%CI: 1.16–74.75), pTNM = II group (OS, HR = 2.31, 95%CI: 1.10–4.84; CSS, HR = 2.43, 95%CI: 1.15–5.12), and group who received postoperative chemotherapy (OS, HR = 4.15, 95%CI: 1.86–9.27; CSS, HR = 5.05, 95%CI: 2.16–11.79) alone, the ACCI was significantly associated with the prognosis.

## 4. Discussion

In this hospital-based retrospective cohort study, we mainly aimed to explore the ability of ACCI to predict the postoperative prognosis of ESCC patients after radical esophagectomy. Our results showed that a higher ACCI, larger tumor size, and advanced pTNM stage were potential factors predictive of a worse prognosis, while the use of adjuvant chemotherapy was associated with a better prognosis.

The results of the current study indicate that the ACCI was negatively associated with the OS and CSS among patients with esophageal cancer, which is in line with previous studies. In a study conducted by Aoyama et al., a total of 122 patients who underwent curative surgery followed by adjuvant chemotherapy for esophageal cancer were included. An ACCI of 5 was regarded as the optimal critical point of classification, considering the survival rates. The multivariate analysis demonstrated that the ACCI was a significant independent risk factor for both the OS (HR = 1.930, 95%CI: 1.126–3.313) and recurrence-free survival (HR = 2.241, 95%CI: 1.375–3.651) [16]. A similar association was also observed between the ACCI and other cancers. Takada et al. reported that the 5-year overall survival rate in an ACCI ≥ 5 group (73.5%) was significantly lower than that of an ACCI < 5 group (96.6%) (*p* < 0.001) among patients with ampullary tumors [27]. Qu et al. conducted research on the survival of 268 intrahepatic cholangiocarcinoma patients after curative resection. This study also found that higher ACCI scores (ACCI ≥ 4) were associated with a poorer OS (HR = 1.134, 95% CI: 1.015–1.267, *p* = 0.026) [28]. It should be mentioned that the ACCI grouping cut-off value in the current study was slightly different from those of these two studies. In the study of Takada et al., the receiver operating characteristic (ROC) curve was applied to gain the cut-off value (4 points), but in the other study by Qu et al., the methodology was not mentioned [27,28]. In our current study, the X-tile software was utilized to calculate the optimal cut-off value. X-tile is a type of software that is useful for dealing with selection complexity based on the time-dependent assessment of the outcome, and it has been used in several cancer studies [29,30]. The establishment of the optimal cut-off value is necessary to predict the prognosis of esophageal cancer.

Tumor size has frequently been suggested to be an important predictive factor for cancer survival. A previous study proved that a larger tumor size in the case of esophageal cancer is correlated with a higher risk of cancer-specific mortality [31]. However, the optimal cut-off value of the tumor size is still under discussion. Previous studies used a range of 3.0–5.0 cm of the tumor size to categorize patients [32,33,34]. Worldwide, the pTNM stage is one of the most common staging systems used for postoperative cancer patients, and it has been recognized as a traditional prognosis predictor of many cancers, including ESCC [35,36,37]. Our results further imply that the pTNM might be the strongest factor in predicting the prognosis among all the studied factors. In further subgroup analyses, we found that ACCI could act as a significant prognostic factor only for ESCC patients with a mild disease severity, i.e., those who had smaller tumor sizes or early pTNM stages. These results imply that ACCI might be particularly useful for predicting the prognosis of cancer patients with a mild disease severity.

Furthermore, chemotherapy is also an important factor affecting the prognosis of cancer patients. Postoperative adjuvant chemotherapy is considered to contribute to the survival of esophageal cancer patients [38]. Our research also proved the beneficial effects of adjuvant chemotherapy on both the OS and CSS.

There is no doubt that the interpretation of the current study’s results should be performed cautiously due to several study limitations. Firstly, the patients were recruited from a single hospital. Thus, selection bias could not be avoided, and the generalization is limited. Secondly, only a small sample size of the study population was included in the current study, which might have caused a lack of statistical power in the case of certain subgroup analyses. Finally, the median follow-up time was short, which may have affected the survival events, especially death due to causes other than esophageal cancers. Therefore, future multi-center studies with larger sample sizes and longer follow-up times are warranted to confirm the predictive ability of ACCI for patients with esophageal cancer.

## 5. Conclusions

In conclusion, the current study showed that the ACCI is an independent prognostic factor for the long-term prognosis of patients after radical esophagectomy. The use of the ACCI might be integrated with the use of TNM staging and tumor size to better predict the prognosis of patients with esophageal cancer, especially those with a mild disease severity. We believe that the ACCI can be used as a marker to guide treatment decisions for patients with esophageal cancer who also have comorbidities.

## Figures and Tables

**Table 1:** Comorbidity distribution based on the Charlson comorbidity index (n = 352).

Comorbidity	Score	n	(%)
Myocardial infarction	1	1	0.3%
Congestive heart failure	1	3	0.9%
Peripheral vascular disease	1	100	28.4%
Cerebrovascular disease	1	36	10.2%
Dementia	1	1	0.3%
Chronic pulmonary disease	1	135	38.4%
Rheumatic disease	1	0	0.0%
Peptic ulcer disease	1	18	5.1%
Mild liver disease	1	65	18.5%
Diabetes mellitus without end-organ damage	1	21	6.0%
Diabetes mellitus with end-organ damage	2	1	0.3%
Hemiplegia	2	0	0.0%
Renal disease	2	3	0.9%
Any malignancy *	2	0	0.0%
Lymphoma	2	0	0.0%
Leukemia	2	0	0.0%
Moderate liver disease	3	9	2.6%
Metastatic solid tumor	6	0	0.0%
Acquired immunodeficiency syndrome (AIDS)	6	0	0.0%

* Excluding esophageal cancer.

**Table 2:** Comparison of the clinical variables between esophageal cancer patients in different ACCI groups.

Variables	All Patients (n = 352)	ACCI < 5 Group (n = 300)	ACCI ≥ 5 Group (n = 52)	*p* Value
Age, median (IQR), years	63.00 (56.00–68.00)	61.00 (55.00–75.00)	72.00 (65.25–76.00)	<0.001
Age group				<0.001
<65 years	225 (63.9%)	212 (70.7%)	13 (25.0%)	
≥65 years	127 (36.1%)	88 (29.3%)	39 (75.0%)	
Gender				0.222
Female	91 (25.9%)	74 (24.7%)	17 (32.7%)	
Male	261 (74.1%)	226 (75.3%)	35 (67.3%)	
Tumor size, median (IQR), cm	4.25 (3.00–6.00)	4.00 (3.00–5.00)	3.9 (3.00–5.00)	0.172
pTNM stage				0.619
I	82 (23.3%)	72 (24.0%)	10 (19.2%)	
II	121 (34.4%)	104 (34.7%)	17 (32.7%)	
III	149 (42.3%)	124 (41.3%)	25 (48.1%)	
Degree of differentiation				0.056
High/Medium	229 (65.1%)	190 (63.3%)	39 (75.0%)	
Poor	92 (26.1%)	84 (28.0%)	8 (15.4%)	
Postoperative chemotherapy				0.771
No	243 (69.0%)	208 (69.3%)	35 (67.3%)	
Yes	109 (31.0%)	92 (30.7%)	17 (32.7%)	
Postoperative complications				0.117
No	143 (40.6%)	127 (42.3%)	16 (30.8%)	
Yes	209 (59.4%)	173 (57.7%)	36 (69.2%)	
Follow-up time, median (IQR, months)	23.37 (14.08–33.45)	23.35 (14.55–33.12)	23.60 (11.27–35.27)	0.488

**Table 3:** Cox proportional hazard analysis of the prognostic predictors associated with overall survival among patients with esophageal squamous cell carcinoma (n = 352).

		Univariate Analysis		Multivariate Analysis	
Variables	Survival/Total (%)	HR (95%CI)	*p* Value	HR * (95%CI)	*p* Value
Age group, years					
<65	157 (69.8%)	Reference			
≥65	77 (60.6%)	1.43 (0.99–2.06)	0.057		
Gender					
Female	63 (69.2%)	Reference		Reference	
Male	171 (65.5%)	1.32 (0.86–2.03)	0.200	1.26 (0.80–1.99)	0.326
ACCI					
<5	209 (69.7%)	Reference		Reference	
≥5	25 (48.1%)	1.74 (1.13–2.68)	0.011	1.63 (1.04–2.56)	0.035
Tumor size, cm					
<3.30	102 (79.7%)	Reference		Reference	
≥3.30	123 (57.2%)	2.79 (1.80–4.33)	<0.001	1.67 (1.05–2.66)	0.032
pTNM					
I	77 (93.9%)	Reference		Reference	
II	78 (64.5%)	6.50 (2.57–16.41)	<0.001	4.74 (1.82–12.32)	0.001
III	79 (53.0%)	9.75 (3.94–24.18)	<0.001	6.08 (2.37–15.60)	<0.001
Degree of differentiation					
High/Medium	147 (64.2%)	Reference		Reference	
Poor	60 (65.2%)	0.94 (0.63–1.42)	0.644	1.04 (0.69–1.58)	0.835
Postoperative chemotherapy					
No	165 (67.9%)	Reference		Reference	
Yes	69 (63.3%)	0.98 (0.80–1.18)	0.787	0.60 (0.40–0.91)	0.015
Postoperative complications					
No	112 (78.3%)	Reference		Reference	
Yes	122 (58.4%)	1.49 (0.98–2.27)	0.062	1.32 (0.85–2.05)	0.224

* Adjusted for gender, ACCI, tumor size, pTNM, degree of differentiation, postoperative chemotherapy, and postoperative complications.

**Table 4:** Cox proportional hazard analysis of the prognostic predictors associated with cancer specific survival among patients with esophageal squamous cell carcinoma (n = 343).

		Univariate Analysis		Multivariate Analysis	
Variables	Survival/Total (%)	HR (95%CI)	*p* Value	HR * (95%CI)	*p* Value
Age group, years					
<65	157 (71.4%)	Reference			
≥65	77 (62.6%)	1.43 (0.98–2.09)	0.067		
Gender					
Female	63 (71.6%)	Reference		Reference	
Male	171 (67,1%)	1.39 (0.89–2.17)	0.154	1.28 (0.80–2.05)	0.311
ACCI					
<5	209 (71.1%)	Reference		Reference	
≥5	25 (51.0%)	1.78 (1.13–2.80)	0.013	1.75 (1.08–2.82)	0.022
Tumor size, cm					
<3.30	102 (80.3%)	Reference		Reference	
≥3.30	123 (59.4%)	2.70 (1.72–4.23)	<0.001	1.66 (1.03–2.69)	0.039
pTNM					
I	77 (93.9%)	Reference		Reference	
II	78 (66.1%)	6.16 (2.43–15.62)	<0.001	4.70 (1.80–12.23)	0.002
III	79 (55.2%)	9.28 (3.71–22.93)	<0.001	5.99 (2.33–15.40)	<0.000
Degree of differentiation					
High/Medium	147 (65.6%)	Reference		Reference	
Poor	60 (66.7%)	0.96 (0.63–1.47)	0.853	1.17 (0.75–1.80)	0.494
Postoperative chemotherapy					
No	165 (69.0%)	Reference		Reference	
Yes	69 (66.3%)	0.89 (0.60–1.33)	0.573	0.57 (0.37–0.87)	0.009
Postoperative complications					
No	112 (78.3%)	Reference		Reference	
Yes	122 (61.0%)	1.40 (0.91–2.14)	0.125	1.30 (0.83–2.03)	0.256

* Adjusted for gender, ACCI, tumor size, pTNM, degree of differentiation, postoperative chemotherapy, and postoperative complications.

## Data Availability

Not applicable.

## Supplementary Materials

The following supporting information can be downloaded at: https://www.mdpi.com/article/10.3390/jcm11226737/s1, Table S1: Interactions between the prognostic predictors of overall survival among patients with esophageal cancer (n = 352); Table S2: Interactions between the prognostic predictors of cancer-specific survival among patients with esophageal cancer (n = 343); Figure S1: Subgroup analysis of ACCI hazard ratio plot of overall survival; Figure S2: Subgroup analysis of ACCI hazard ratio plot of cancer-specific survival.
