# Supplementary material for: Effect of the Age-Adjusted Charlson Comorbidity Index on the Survival of Esophageal Squamous Cell Carcinoma Patients after Radical Esophagectomy
Source: J Clin Med. 2022 Nov 14;11(22):6737. doi: 10.3390/jcm11226737 (PMC9696569; doi:10.3390/jcm11226737)
Supplement: Supplementary file 1 [file jcm-11-06737-s001.zip › jcm-1995421-supplementary.pdf]

Supplementary Material

Supplementary Table S1. Interactions of Prognostic predictors for overall survival among patients with esophageal cancer (n=352).

							95.0% CI	
Model	B	SE	Wald	df	P	HR	Lower	Upper
ACCI*Tumor size#	-1.153	0.516	4.999	1	0.025	0.32	0.12	0.87
ACCI group*pTNM#			5.078	2	0.079			
ACCI group*pTNM(1) #	-1.261	0.996	1.603	1	0.205	0.28	0.04	2.00
ACCI group*pTNM(2) #	-1.948	0.978	3.969	1	0.046	0.14	0.02	0.97
ACCI group*Postoperative chemotherapy#	1.128	0.477	5.599	1	0.018	3.09	1.21	7.87

#Adjusted to gender, ACCI, tumor size, pTNM, degree of differentiation, postoperative chemotherapy, and postoperative complications.

Supplementary Table S2. Interactions of Prognostic predictors for cancer-specific survival among patients with esophageal cancer (n=343).

							95.0% CI	
Model	B	SE	Wald	df	P	HR	Lower	Upper
ACCI*Tumor size#	-1.099	0.545	4.065	1	0.044	0.33	0.11	0.97
ACCI group*pTNM#			4.804	2	0.091			
ACCI group*pTNM(1) #	-1.282	0.999	1.646	1	0.200	0.28	0.04	1.97
ACCI group*pTNM(2) #	-1.962	0.987	3.951	1	0.047	0.14	0.02	0.97
ACCI group*Postoperative chemotherapy#	1.154	0.498	5.366	1	0.021	3.17	1.19	8.42

#Adjusted to gender, ACCI, tumor size, pTNM, degree of differentiation, postoperative chemotherapy, and postoperative complications

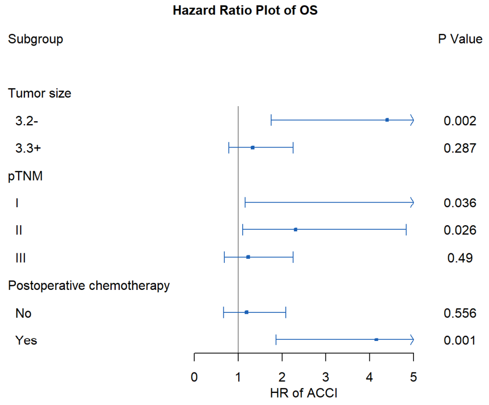

Supplementary Figure S1. Subgroup analysis of ACCI hazard ratio plot of overall survival.

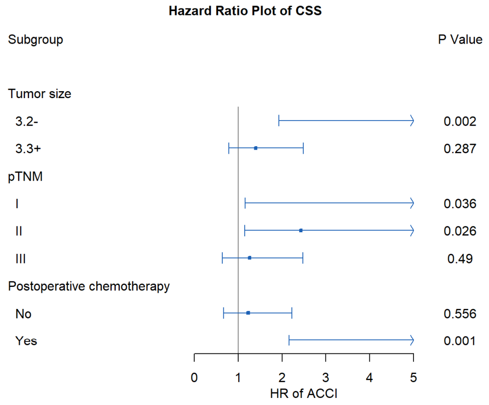

Supplementary Figure S2. Subgroup analysis of ACCI hazard ratio plot of cancer-specific survival.
